# Analysis of the Number of Euploid Embryos in Preimplantation Genetic Testing Cycles With Early-Follicular Phase Long-Acting Gonadotropin-Releasing Hormone Agonist Long Protocol

**DOI:** 10.3389/fendo.2020.00424

**Published:** 2020-07-21

**Authors:** Gang Li, Yifang Wu, Wenbin Niu, Jiawei Xu, Linli Hu, Hao Shi, Yingpu Sun

**Affiliations:** Center for Reproductive Medicine, The First Affiliated Hospital of Zhengzhou University, and Henan Province Key Laboratory of Reproduction and Genetics, Zhengzhou, China

**Keywords:** controlled ovarian stimulation, early-follicular short-acting GnRH agonist long protocol (EFLL), midluteal short-acting GnRH agonist long protocol (MLSL), euploidy, preimplantation genetic testing (PGT)

## Abstract

Studies have shown that early-follicular phase long-acting gonadotropin-releasing hormone (GnRH) agonist long protocol (EFLL), a popular controlled ovarian hyperstimulation protocol widely used in China, leads to higher rates of implantation and clinical pregnancy, as well as lower rates of spontaneous abortion and ectopic pregnancy in patients undergoing *in vitro* fertilization treatment. However, the impact of EFLL on euploid embryos and its underlying mechanisms remain unclear. To address these gaps of knowledge, we conducted a retrospective comparative study of 310 preimplantation genetic testing (PGT) cycles with a total of 1,541 embryos using the EFLL protocol or midluteal short-acting GnRH agonist long protocol (MLSL). Patients were matched by PGT subtype [aneuploidies (PGT-A) vs. PGT for chromosomal structural rearrangements (PGT-SR)], age (±2 years), and body mass index (±1 kg/m^2^). For PGT-A, there was no significant difference in the number of euploid embryos (1.80 ± 1.47 for EFLL vs. 1.84 ± 2.03 for MLSL, *p* > 0.05) or the rate of euploidy (44.6 vs. 36.9%, *p* > 0.05). For PGT-SR, the number of euploid embryos in the EFLL group was significantly higher than that in the MLSL group (1.76 ± 1.54 vs. 1.21 ± 1.24, *p* < 0.05). A higher euploidy rate was also observed with the EFLL protocol compared with that obtained in MLSL (31.9 vs. 25.7%), although the difference was not statistically significant (*p* > 0.05). Compared with the MLSL protocol, more euploid embryos were achieved when using the EFLL protocol in PGT-SR, demonstrating the value in PGT-SR. To the best of our knowledge, this study is the first one to compare embryonic outcomes between EFLL and MLSL, providing key insights into the clinical application of EFLL in PGT cycles. In the light of the limited sample size of our study, we recommend that these questions be explored using a larger prospective study.

## Introduction

Preimplantation genetic testing (PGT), including aneuploidies (PGT-A), and PGT for chromosomal structural rearrangements (PGT-SR), is a set of advanced clinical procedures (1) in the area of assisted reproductive technology (ART). The use of PGT substantially reduces the proportion of implantation and pregnancy failures related to aneuploidies (2). Extensive research into aneuploidy has been performed, and the associated risk factors and mechanisms have been reported in detail (3, 4). Among identified risk factors, advanced maternal age is associated with lower rates of embryonic euploidy (5, 6). Studies (7) exploring the relationship between body mass index (BMI) and euploidy have yielded inconclusive results, and some degree of controversy remains. In addition, laboratory procedures, including culture conditions and blastocyst morphology, can affect the rate of aneuploidy (8, 9).

For patients who have undergone PGT cycles, euploid embryos may be precious, especially for those with advanced maternal age, unexplained recurrent miscarriage, recurrent *in vitro* fertilization (IVF) failure, or chromosomal abnormalities. While the number of euploid embryos is extremely important, improved outcomes may be obtained with a well-developed euploid embryo even if there is only one embryo. It has been noted that numerical chromosomal aneuploidy (36.4%, 4/11) is present by analyzing the natural cycle in young woman (10). The influence of controlled ovarian stimulation is of paramount importance, and three protocols are currently used for PGT: the midluteal short-acting gonadotropin-releasing hormone (GnRH) agonist long protocol (MLSL), the GnRH antagonist protocol, and the microstimulation protocol. Although some studies have found that moderate ovarian stimulation does not significantly decrease the rate of embryo euploidy (11), the optimal protocol remains to be identified. It has been suggested that the GnRH antagonist protocol results in a higher number of euploid embryos compared with the GnRH agonist (GnRH-a) protocol (12). The early-follicular long-acting GnRH-a long protocol (EFLL) has become increasingly popular in China because it is associated with improved endometrial receptivity, higher implantation rates and clinical pregnancy rates, and lower spontaneous abortion rates (13). Data from our center on 5,197 IVF/ICSI cycles from May 2015 to 2016 showed better outcomes with the EFLL protocol than that with the MLSL protocol: higher implantation rate (45.34 vs. 37.68%) and clinical pregnancy rate (63.72 vs. 52.67%), lower miscarriage rate (8.41 vs. 11.55%), and lower ectopic pregnancy rate (1.52 vs. 3.30%) (14). Ron-El et al. (15) have suggested that the metaphase II (MII) oocyte rate and successful embryo rate are significantly enhanced with the EFLL protocol compared with that obtained with MLSL. It had been shown that oocytes and embryos have comparable developmental potency whether stimulation is begun in the luteal phase or the follicular phase (16). However, the impact of EFLL on the rate of euploid embryos remains unclear.

The aim of study was to determine which PGT protocol is associated with optimal embryonic quality and higher number of available embryos. To this end, the number of euploid embryos obtained and the euploidy rates between the EFLL and MLSL protocols were compared in a cohort of Chinese women undergoing PGT.

## Materials and Methods

### Study Design and Settings

Data in this retrospective comparative study were from the Clinical Reproductive Medicine Management System/Electronic Medical Record Cohort Database at the Reproductive Medical Center, First Affiliated Hospital of Zhengzhou University. Data on patient demographics, ART history, protocol used, and embryonic outcomes were collected for 2,616 PGT cycles performed between December 2010 and May 2019. This study was approved by the Ethics Committee of the Reproductive Medicine Centre, First Affiliated Hospital of Zhengzhou University, China. Written informed consent was provided by all study participants.

### Participants

The EFLL protocol and MLSL protocol were selected from PGT cycles using the EFLL or MLSL protocol between July 2015 and May 2019. Each patient was identified by their unique medical record number. Only one cycle per patient was included in order to avoid confounding by patient-specific factors. This yielded 1,564 eligible cycles to be included in the study.

To better know the impact of EFLL protocol on euploid embryos, we first classified PGT cycles as PGT-A or PGT-SR. In the comparison part, each cycle using the EFLL protocol was matched with four cycles with the MLSL protocol by age (±2 years) and BMI (±1 kg/m^2^).

### Ovarian Stimulation Protocols

All patients underwent appropriate controlled ovarian stimulation protocols by experienced physicians according to the patient's baseline characteristics and ART history. For patients treated with the EFLL protocol, we administrated 3.75 mg of long-acting GnRH-a (Diphereline, Ipsen, France) on days 2–4 of menstruation. Patients were monitored with ultrasound, and serum sex hormones levels were tracked. Down-regulation standard was classified as follows: (a) serum luteinizing hormone (LH) of <5 IU/L, follicle-stimulating hormone (FSH) of <5 IU/L, estradiol (E_2_) of <30 μg/mL, progesterone (P) of <1 ng/mL; (b) no functional cyst, follicle size 3–5 mm under ultrasound, ovulation induced. The initial dose of gonadotropin (Gn) was determined based on the patient's antral follicle count (AFC), age, BMI, and previous ovarian response to stimulation. The dosage was then continuously adjusted according to the patient's response. When dominant follicles measuring >16 mm accounted for 60% of follicles or a follicle reached 20 mm in mean diameter, trigger was performed using 250 μg of recombinant human chorionic gonadotropin (r-hCG, Merck Serono, Geneva, Switzerland) in combination with 2,000 IU u-HCG (Livzon, Guangzhou, China). Oocyte retrieval under the guidance of transvaginal ultrasound was performed 37 h after trigger.

For patients treated with the MLSL protocol, short-acting GnRH-a (decapeptyl, Ferring, Switzerland) at a dose of 0.1 mg was administered during the midluteal phase. After 7 days, patients were monitored to ensure no functional cyst and negative hCG test. Medication was continued for 3 days; after that, the dosage reduced to 0.05 mg for a further 4 days. The pituitary down-regulation standard and trigger injection were identical to those used for the EFLL protocol.

### Laboratory Procedures

Intracytoplasmic sperm injection was used in 4 to 6 h after retrieval for all cycles. Embryos were cultured to blastocyst stage using Vitrolife (Goteborg, Sweden) sequential medium, and the culture environment was 37°C, 6% CO_2_, 5% O_2_, and 89% N_2_. The evaluation was under grading criteria (17) on the morning of day 5 or 6. Only expansion degree 4–6 and blastocysts with inner cell mass/trophectoderm scores AA, BA, AB, and BB were defined as high-quality. The experienced embryologists scored blastocysts under consistent laboratory conditions, and high-quality blastocysts were trophectoderm biopsied using the laser method.

All blastocysts were genetically tested for aneuploidy using array comparative genomic hybridization for comprehensive chromosomal screening with diagnosis performed by experienced technicians. The amplified samples were labeled using the fluorescent labeling system (Illumina, San Diego, CA, USA). After labeling and washing, the scanned images were generated and analyzed using BlueFuse Multi software (BlueGnome, Cambridge, UK). Results of PGT were recorded as normal/euploidy, abnormal/aneuploidy, or no signal. Aneuploid embryos included those with the loss of a chromosome or having an extra full chromosome or segmental abnormality, those with a chromosome piece (at least 6 MB in size) missing or added, and mosaicism if there was a mixture of euploidy and aneuploidy. A small minority (42/1,541) of cases were diagnosed as no signal for amplification failure. Normal/euploid embryos were frozen and selected for transfer in a subsequent thaw embryo transfer cycle.

### Statistical Analysis

The primary outcome in the retrospective comparative study was the number of euploid embryos and the euploidy rate. The secondary outcomes included the number of oocytes retrieved. Continuous variables were represented as mean ± standard deviation. Two-sample *t*-tests/non-parametric analysis of variance were used to compare continuous variables and Fisher exact test/χ^2^ analysis were used to compare euploidy rate between study groups. Statistical significance was defined at an α level of < 0.05. All analyses were carried out using SPSS version 25 (Chicago, IL, USA).

## Results

A total of 310 cycles were performed between July 2015 and May 2019, of which 62 applied the EFLL protocol and 248 applied the MLSL protocol. There were 125 PGT-A cycles and 185 PGT-SR cycles (31 Robertsonian translocations, 131 reciprocal translocations, and 23 inversions). A total of 1,541 blastocysts were biopsied and screened, of which 473 (30.69%) yielded euploid embryos, 980 (63.59%) were aneuploid embryos, 46 (2.99%) were mosaic embryos, and 42 (2.73%) screened as no signal. For PGT-A, there were 613 blastocysts, of which 229 (37.36%) were euploid embryos, 353 (57.59%) were aneuploid embryos, 17 (2.77%) were mosaic embryos, and 14 (2.28%) screened as no signal. For PGT-SR, there were 928 blastocysts, of which 244 (26.29%) were euploid embryos, 627 (67.56%) were aneuploid embryos, 29 (3.13%) were mosaic embryos, and 28 (3.02%) screened as no signal. Embryos screened as no signal were excluded in the analysis. For PGT-A, there were 5 (4.95%) mosaic embryos in the EFLL group and 12 (2.41%) in the MLSL group, respectively. For PGT-SR, there were 11 (5.39%) mosaic embryos in the EFLL group and 18 (2.58%) in the MLSL group.

Baseline characteristics for PGT-A cycles (*n* = 125) and PGT-SR cycles (*n* = 185) are shown in Tables 1, 2, respectively. No significant differences were observed in AFC, basal FSH, basal E_2_, or basal LH between the PGT-A and PGT-SR groups.

**Table 1:** Baseline characteristics and outcomes of PGT-A cycle.

**Characteristics**	**EFLL (*n* = 25)**	**MLSL (*n* = 100)**	***P*-value**
Age (years)	31.16 ± 5.80	31.20 ± 5.63	NS
BMI (kg/m^2^)	21.83 ± 1.75	22.09 ± 2.36	NS
AFC	14.40 ± 6.60	15.24 ± 5.44	NS
bFSH (IU/L)	6.66 ± 1.560	6.51 ± 1.52	NS
bE_2_ (pg/mL)	51.58 ± 47.58	51.62 ± 158.06	NS
bLH (IU/L)	4.92 ± 2.22	5.30 ± 2.83	NS
Gn days	13.48 ± 2.42	10.53 ± 1.43	<0.001
Total dosage of Gn (IU)	2,654.00 ± 919.00	2,268.13 ± 775.03	0.034
Endometria thickness on hCG day (mm)	11.20 ± 2.90	11.38 ± 3.05	NS
LH on hCG day (IU/L)	0.72 ± 0.54	1.56 ± 0.78	<0.001
E_2_ on hCG day (pg/mL)	4,207.56 ± 2,069.66	5,493.75 ± 2,426.99	0.016
No. of retrieved oocytes	15.64 ± 4.93	15.81 ± 7.19	NS
E_2_/no. of retrieved oocytes	276.12 ± 109.70	381.35 ± 169.41	0.004
MII oocytes rate (%)	80.1% (313/391)	85.2% (1,347/1,581)	NS
Fertilization rate (%)	80.2% (251/313)	77.3% (1,041/1,347)	NS
No. of biopsied blastocysts	4.16 ± 2.39	5.09 ± 3.85	NS
No. of euploid embryos	1.80 ± 1.47	1.84 ± 2.03	NS
Euploidy rate (%)	44.6% (45/101)	36.9% (184/498)	NS

*BMI, body mass index; AFC, antral follicle count; Gn, gonadotropin; hCG, human chorionic gonadotrophin*.

*Data are presented as mean ± SD or % (no./total no.)*.

**Table 2:** Baseline characteristics and outcomes of PGT-SR cycle.

**Characteristics**	**EFLL (*n* = 37)**	**MLSL (*n* = 148)**	***P-*value**
**Classification**
Robertsonian translocation	18.9% (7/37)	16.2% (24/148)	
Reciprocal translocation	59.5% (22/37)	73.6% (109/148)	
Inversion	21.6% (8/37)	10.1% (15/148)	
Age (years)	29.78 ± 4.95	29.64 ± 5.16	NS
BMI (kg/m^2^)	22.19 ± 2.26	22.14 ± 2.14	NS
AFC	15.46 ± 6.56	15.18 ± 6.62	NS
bFSH (IU/L)	6.13 ± 1.87	6.50 ± 1.92	NS
bE_2_ (pg/mL)	37.96 ± 25.64	45.30 ± 52.67	NS
bLH (IU/L)	4.77 ± 2.55	5.35 ± 3.15	NS
Gn days	12.43 ± 2.50	10.87 ± 1.51	0.001
Total dosage of Gn (IU)	2,351.69 ± 1,003.72	2,218.07 ± 822.70	NS
Endometria thickness on hCG day (mm)	12.22 ± 2.57	11.30 ± 2.56	NS
LH on hCG day (IU/L)	0.69 ± 0.47	1.55 ± 0.93	<0.001
E_2_ on hCG day (pg/mL)	5,415.65 ± 2,849.30	5,650.79 ± 3,432.62	NS
No. of retrieved oocytes	19.35 ± 9.01	16.61 ± 8.79	NS
E_2_/no. of retrieved oocytes	283.78 ± 126.87	377.20 ± 259.45	0.035
MII oocytes rate (%)	80.9% (579/716)	82.4% (2,026/2,458)	NS
Fertilization rate (%)	81.2% (470/579)	76.6% (1,551/2,026)	0.019
No. of biopsied blastocysts	5.89 ± 3.96	4.80 ± 3.26	NS
No. of euploid embryos	1.76 ± 1.54	1.21 ± 1.24	0.024
Euploidy rate (%)	31.9% (65/204)	25.7% (179/696)	NS

*BMI, body mass index; AFC, antral follicle count; Gn, gonadotropin; hCG, human chorionic gonadotrophin*.

*Data are presented as mean ± SD or % (no./total no.)*.

**Figure 1:**
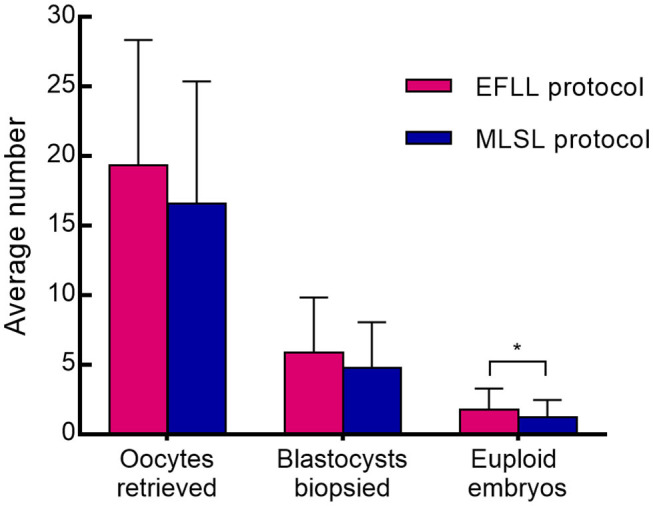
Oocytes retrieved, blastocysts biopsied, and embryos between EFLL and MLSL protocol for PGT-SR. Oocytes retrieved, blastocysts biopsied, and embryos diagnosed as being chromosomally euploidy from patients who underwent PGT-SR cycles using the EFLL protocol and the MLSL protocol, respectively. Values are presented as mean ± standard deviation. *p < 0.05.

Patients treated with the EFLL protocol for PGT-A had a higher number of Gn days and total Gn dosage compared with those treated with the MLSL protocol (13.48 ± 2.42 days, 2,654.00 ± 919.00 IU for EFLL protocol; 10.53 ± 1.43 days, 2,268.13 ± 775.03 IU for MLSL protocol, *p* <0.05 for both comparisons). A similar pattern was observed for PGT-SR cycles (12.43 ± 2.50 days, 2,351.69 ± 1,003.72 IU for EFLL protocol; 10.87 ± 1.51 days, 2,218.07 ± 822.70 IU for MLSL protocol, *p* < 0.05 for Gn-days comparisons). However, there were no differences in total Gn dosage between the groups (2,351.69 ± 1,003.72 vs. 2,218.07 ± 822.70 IU, *p* > 0.05). The level of LH on hCG administration day was significantly lower for the EFLL protocol than for MLSL (PGT-A: 0.72 ± 0.54 vs. 1.56 ± 0.78 IU/L; PGT-SR: 0.69 ± 0.47 vs. 1.55 ± 0.93 IU/L; *p* < 0.05 for both comparisons). For PGT-A, the level of E_2_ was significantly lower for the EFLL protocol (4,082.99 ± 2,225.98 vs. 5,413.14 ± 2,537.95 pg/mL, *p* < 0.05). For PGT-SR, the level of E_2_ was lower for the EFLL protocol, although the difference was not significant (5,415.65 ± 2,849.30 vs. 5,642.67 ± 3,466.10 pg/mL, *p* > 0.05). The ratio of E_2_ to the number of retrieved oocytes was significantly lower for the EFLL protocol in PGT (PGT-A: 276.12 ± 109.70 vs. 381.35 ± 169.41 pg/mL; PGT-SR: 283.78 ± 126.87 vs. 377.20 ± 259.45 pg/mL; *p* < 0.05 for both comparisons). Embryonic outcomes did not differ significantly between the two groups for PGT-A (number of euploid embryos: 1.80 ± 1.47 vs. 1.84 ± 2.03; euploidy rate: 44.6 vs. 36.9%; *p* > 0.05 for both comparisons). However, the fertilization rate and the number of euploid embryos were higher for the EFLL protocol than for the MLSL protocol for PGT-SR (81.2 vs. 76.6%, *p* > 0.05; 1.76 ± 1.54 vs. 1.21 ± 1.24, *p* < 0.05). The number of oocytes retrieved, blastocysts biopsied, and euploid embryos were higher in patients treated with the EFLL protocol than in those treated with the MLSL protocol (Figure 1).

Outcomes according to type of rearrangement between study groups in PGT-SR cycle are shown in Table 3. The reciprocal translocations in the MLSL group were higher than those in the EFLL group (73.6 vs. 59.5%), whereas the inversions were overrepresented in the EFLL group (21.6 vs. 10.1%). The euploidy rate was higher in the EFLL group in Robertsonian translocation and reciprocal translocation, but it was higher in the MLSL group in inversions (Robertsonian translocation: 45.2 vs. 40.0%; reciprocal translocation: 26.2 vs. 21.3%; inversion: 38.3 vs. 41.3%, *p* > 0.05).

**Table 3:** Outcomes according to type of rearrangement in PGT-SR cycle.

	**EFLL (*****n*** **=** **37)**			**MLSL (*****n*** **=** **148)**		
	**Robertsonian translocation**	**Reciprocal translocation**	**Inversion**	**Robertsonian translocation**	**Reciprocal translocation**	**Inversion**
Cycles	7 (18.9%)	22 (59.5%)	8 (21.6%)	24 (16.2%)	109 (73.6%)	15 (10.1%)
Age (years)	30.00 ± 5.66	29.68 ± 4.62	29.87 ± 5.84	30.63 ± 5.06	29.12 ± 4.62	31.80 ± 4.35
BMI (kg/m^2^)	21.76 ± 2.94	22.35 ± 1.96	22.10 ± 2.67	22.03 ± 2.13	22.12 ± 2.06	22.41 ± 2.80
No. of retrieved oocytes	19.43 ± 12.42	19.36 ± 7.93	19.25 ± 9.85	14.83 ± 7.64	17.04 ± 8.64	16.33 ± 11.50
MII oocytes rate (%)	76.5% (104/136)	83.1% (354/426)	78.6% (121/154)^a^	82.0% (292/356)	82.0% (1,523/1,857)	86.1% (211/245)^a^
Fertilization rate (%)	76.9% (80/104)	83.3% (295/354)^b^	78.5% (95/121)	66.8% (195/292)^cd^	78.1% (1,190/1,523)^bc^	78.7% (166/211)^d^
No. of biopsied blastocysts	5.00 ± 3.42^e^	6.09 ± 4.46^fg^	6.13 ± 3.18^eg^	3.46 ± 2.34^h^	5.00 ± 3.35^fh^	5.47 ± 3.52
No. of euploid embryos	2.00 ± 2.16^i^	1.50 ± 1.14	2.25 ± 1.90	1.33 ± 1.13^i^	1.05 ± 1.09	2.20 ± 1.90
Euploidy rate (%)	45.2% (14/31)^j^	26.2% (33/126)^j^	38.3% (18/47)	40.0% (32/80)^k^	21.3% (114/536)^kl^	41.3% (33/80)^l^

*BMI, body mass index*.

*Data are presented as mean ± SD or % (no./total no.)*.

a−l *p < 0.05, for analysis of variance comparison/Fisher exact test/χ^2^-test between columns in the same row*.

## Discussion

Preimplantation genetic testing can select embryos with euploid karyotypes to reassure and improve IVF outcomes, especially for patients who are inherently predisposed to genetic disorder and transmitted to their offspring. It has been suggested that a higher number of euploid embryos attracted relating to a higher ovarian response (18) and oocyte yield is also an important predictor for successful PGT outcome. Therefore, a suitable controlled ovarian stimulation protocol is used for obtaining a relatively maximum ovarian response in PGT cycles to ensure that at least one euploid embryo is available for transfer.

Over the last 5 years, the EFLL protocol has been increasingly used in China for IVF patients because of its potential to yield higher pregnancy and live-birth rates (13, 14, 19, 20). The success of this protocol has been attributed to its improvement of the quality of the endometrium or the embryo. However, most studies in this area have been limited to examination of endometrial receptivity and the intrauterine microenvironment. Endometrial thickness as measured by ultrasonography has been used to evaluate endometrial receptivity and as a prognostic factor in embryo transplantation (21). Data from several sources suggest that higher endometrial thickness is associated with improved pregnancy rates, independent of the number or quality of embryos (22–24). More recently, this conclusion was confirmed by a retrospective cohort study of 1,512 IVF cycles (25). Also, an endometrial thickness of more than 7 mm was optimal for clinical pregnancy outcomes (26). In our study, endometrial thickness was satisfactory for both protocols and did not vary significantly between them.

Administration of the EFLL protocol improves the ovarian response to exogenous GnRH-a. Effects on the endometrium may be elicited through regulation of enzymes and cytokines. Infiltration of CD68-positive Mvarphi and von Willebrand factor–positive microvessel density were found to be significantly reduced in the GnRH-a treated group (27). In addition, the expression of endometrial αvβ3 vitronectin, interleukin 1 system, and vascular endothelial growth factor was found to be affected by GnRH-a (28, 29).

However, understanding of the role of GnRH-a in embryonic development remains limited. In the present study, the euploidy rate, and the number of euploid embryos were compared between the EFLL and MLSL protocols, with the patient groups matched by PGT-A/SR, age, and BMI. We found no significant differences in the levels of AFC, basal FSH, basal E_2_, or basal LH between the study groups, suggesting similar ovarian function with the two protocols. Our study demonstrates that the EFLL protocol has a beneficial effect in terms of the number of oocytes and euploid embryos for PGT-SR.

Multiple follicle development, along with unusual LH elevation, is found in controlled ovarian stimulation. Early-onset LH peak generally results in early atresia or luteinization of follicles, as well as early meiotic division of oocytes. In our study, deeper pituitary suppression was observed in the EFLL protocol, and the level of LH on hCG administration day was significantly lower than that in the MLSL protocol for PGT. Although the duration and dosage of Gn were elevated when pituitary function was strongly suppressed (30), several studies have found no association between Gn dosage and embryo euploidy (31–33). We speculate that the lower LH levels found with the EFLL protocol and the decreased possibility of premature LH surge reduced the likelihood of abnormal meiotic division of oocytes, leading to a higher euploidy rate.

Hyperphysiological levels of E_2_ may influence oocyte development. Some studies found no association between E_2_ level on hCG administration day and pregnancy outcomes, whereas other studies have reported negative correlations (34–37). Gelety and Buyalos (38) found a lower fertilization rate in cycles with higher E_2_ on hCG compared with the control group (63 ± 4.0% vs. 73 ± 3.0%, *p* < 0.04), supporting a potential adverse effect of E_2_ on oocyte quality. This pattern was also observed in our study. In addition, the ratio of E_2_ to the number of retrieved oocytes in EFLL was lower, tending toward normal physiological levels. Accordingly, the oocyte function or intrafollicular dynamics may be affected, contributing to a higher number of oocytes and embryos.

The number of euploid embryos was significantly higher in our study when using the EFLL protocol in PGT-SR cycles, but not in PGT-A cycles. This finding may stem in part from the higher overall age of patients undergoing PGT-A cycles. Alternatively, the small number of women undergoing PGT-A cycles limited the statistical power to detect differences within this group. However, it is noteworthy that in the PGT-SR cycles there were more oocytes retrieved in the EFLL group (19.35 ± 9.01 vs. 16.61 ± 8.79); thus, the average numbers of fertilized eggs and blastocysts are also higher in the EFLL group than in the MLSL group, which eventually results in more euploid blastocysts. Additionally, the numerically higher euploidy rate in the PGT-SR cycles would seem to be a combined result of the increase in euploid embryos and higher number of embryos biopsied.

Considering the frequency of embryos with an unbalanced karyotype is typically higher in reciprocal than that in Robertsonian translocations and can vary considerably in inversions (39–41), outcomes in three chromosome rearrangement categories from 185 PGT-SR cycles were analyzed. It showed a lower percentage of euploid embryos for reciprocal translocation in both the EFLL group and the MLSL group. Moreover, similar patterns of more oocytes retrieved and euploid embryos were observed in each of the three categories. However, the interchromosomal effect (ICE) may exist in varying degrees depending on the individual rearrangement. Several controversial studies have shown that ICE may present in oocytes (42–44) and cleavage-stage embryos and blastocysts (45, 46), and the occurrence of ICE has related to the region of the translocation and the position of breakpoints (43).

Our study yields valuable insights into PGT cycles and may be used to broaden the application of this treatment, although the mechanisms underlying PGT remain unclear. Our study included a modest sample size and was conducted as a retrospective analysis. Accordingly, larger prospective studies will be needed to confirm our findings and further explain the mechanisms underlying different ovarian stimulation protocols.

To our knowledge, the present study is the first to compare embryonic outcomes between patients treated with EFLL and MLSL protocols. Our findings provide insight into clinical application of the EFLL protocol in PGT cycles.

## Data Availability Statement

The datasets generated for this study are available on request to the corresponding author.

## Ethics Statement

The studies involving human participants were reviewed and approved by the Ethics Committee of Reproductive Medicine Centre, the First Affiliated Hospital of Zhengzhou University, China. The patients/participants provided their written informed consent to participate in this study.

## Author Contributions

GL and YS conceived and designed the experiment. GL and LH selected and supervised suitable patients, recruited the patients, retrieved oocytes, and transferred embryos. WN, JX, and HS performed next generation sequencing and sequencing data analysis. YS provided overall supervision. GL and YW drafted the manuscript. All authors reviewed this manuscript All authors contributed to the article and approved the submitted version.

## Conflict of Interest

The authors declare that the research was conducted in the absence of any commercial or financial relationships that could be construed as a potential conflict of interest.
